# Air Quality Modeling for the Urban Jackson, Mississippi Region Using a High Resolution WRF/Chem Model

**DOI:** 10.3390/ijerph8062470

**Published:** 2011-06-23

**Authors:** Anjaneyulu Yerramilli, Venkata B. Dodla, Srinivas Desamsetti, Srinivas V. Challa, John H. Young, Chuck Patrick, Julius M. Baham, Robert L. Hughes, Sudha Yerramilli, Francis Tuluri, Mark G. Hardy, Shelton J. Swanier

**Affiliations:** 1 Trent Lott Geospatial Visualization Research Centre, Jackson State University, 1230 Raymond Road, Jackson, MS 39204, USA; E-Mails: venkata.b.dodla@jsums.edu (V.B.D.); srinivas.desamsetti@jsums.edu (S.D.); venkatasrinivasc@yahoo.com (S.V.C.); john.h.young@jsums.edu (J.H.Y); cpatrick@jsums.edu (C.P.); julius.m.baham@jsums.edu (J.M.B); robert.l.hughes@jsums.edu (R.L.H.); 3 National Center for Biodefense Communications, Jackson State University, 1230 Raymond Road, Jackson, MS 39204, USA; E-Mail: sudha.yerramilli@jsums.edu; 2 College of Science, Engineering and Technology, Jackson State University, Jackson, MS 39217, USA; E-Mails: francis.tuluri@jsums.edu (F.T.); mark.g.hardy@jsums.edu (M.G.H.); sswanier@jsums.edu (S.J.S.)

**Keywords:** air quality, modeling, WRF/Chem, urban pollution

## Abstract

In this study, an attempt was made to simulate the air quality with reference to ozone over the Jackson (Mississippi) region using an online WRF/Chem (Weather Research and Forecasting–Chemistry) model. The WRF/Chem model has the advantages of the integration of the meteorological and chemistry modules with the same computational grid and same physical parameterizations and includes the feedback between the atmospheric chemistry and physical processes. The model was designed to have three nested domains with the inner-most domain covering the study region with a resolution of 1 km. The model was integrated for 48 hours continuously starting from 0000 UTC of 6 June 2006 and the evolution of surface ozone and other precursor pollutants were analyzed. The model simulated atmospheric flow fields and distributions of NO_2_ and O_3_ were evaluated for each of the three different time periods. The GIS based spatial distribution maps for ozone, its precursors NO, NO_2_, CO and HONO and the back trajectories indicate that all the mobile sources in Jackson, Ridgeland and Madison contributing significantly for their formation. The present study demonstrates the applicability of WRF/Chem model to generate quantitative information at high spatial and temporal resolution for the development of decision support systems for air quality regulatory agencies and health administrators.

## 1. Introduction

As per the EPA urban air quality in the U.S. with reference to ozone will be a growing concern due to its oxidative capacity which will have great impact on the environment in the form of tropospheric ozone formation increases due to photo-chemical activity in presence of sunlight, nitrogen oxides (NO_x_) and volatile organic compounds (VOCs) emitted by cars, power plants, industrial boilers, refineries, chemical plants *etc*, so growing cities are likely to be under great risk in the future. Large numbers of people are likely to be exposed to unhealthy ozone concentrations when ground-level ozone accumulates in urban metropolitan areas under certain weather conditions [1] and quantitative atmospheric dispersion models will be of great help to provide effective decision support systems for planners and administrators.

Air quality concentrations are strongly affected by weather. Developing a basic understanding of how ozone forms will help air quality agencies forecast the effects of weather on ozone. Different scales of weather phenomena are important to air quality. The weather phenomena range from large storm systems that can encompass thousands of kilometers to small turbulent eddies that are a few meters in size. Meteorological conditions that strongly influence air quality include: transport by winds, recirculation of air by local wind patterns, and horizontal dispersion of pollution by wind; variations in sunlight due to clouds and season; vertical mixing and dilution of pollution within the atmospheric boundary layer; temperature; and moisture. The variability of these processes, which affects the pollution variability, is primarily governed by the movement of large-scale high- and low-pressure systems, the diurnal heating and cooling cycles, and local and regional topography. The surface atmospheric flow fields are a resultant of synoptic scale circulations and the local circulations induced by the topography and land use variations. Large-scale winds give the general wind regime and local winds influence the convergence zones. As such, local circulations play an important role in the dispersion of pollutants [2]. A number of studies have indicated the importance of topography and land use variations on the characteristics of pollutants’ dispersion over different regions; e.g., over the Iberian peninsula [3], the Columbia River Basin [4], or the Mexico City basin [5].

Ozone (O_3_) is not emitted directly into the air; instead it forms in the atmosphere as a result of a series of complex chemical reactions between oxides of nitrogen (NO_x_) and hydrocarbons, which together are precursors of ozone. Ozone precursors have both anthropogenic (man-made) and biogenic (natural) origins. Motor vehicle exhaust, industrial emissions, gasoline vapors, and chemical solvents are some of the major sources of NO_x_ and hydrocarbons. Many species of vegetation including trees and plants emit hydrocarbons, and fertilized soils release NO_x_.

In the presence of ultraviolet radiation (*hν*), oxygen (O_2_) and nitrogen dioxide (NO_2_) react in the atmosphere to form ozone and nitric oxide (NO). The resultant ozone reacts with NO to form nitrogen dioxide. A steady state is attained through these reactions:

$$\begin{array}{l} {\text{NO}}_{2}+h\;\text{v}\to \;\;\text{NO}+\text{O}\\ \text{NO}+{\text{NO}}_{2}+{\text{H}}_{2}\text{O}\to \;\;2\text{HONO}\\ \text{HONO}+h\;v\to \;\;\text{NO}+\text{OH}\\ \text{O}+{\text{O}}_{2}\to \;\;{\text{O}}_{3}\\ \text{CO}+2{\text{O}}_{2}\to \;\;{\text{CO}}_{2}+{\text{O}}_{3}\\ {\text{O}}_{3}+\text{NO}\to \;\;{\text{NO}}_{2}+{\text{O}}_{2} \end{array}$$

Even without anthropogenic emissions, these reactions normally result in a natural background ozone concentration of 25 to 45 ppb [6]. Ozone cannot accumulate further unless volatile organic compounds (VOCs), which include hydrocarbons, are present to consume or convert NO back to NO_2_ as:

$$\text{VOC}+\text{NO}\to \;\;\;\;{\text{NO}}_{2}+\text{other products}$$

This equation is a simplified version of many complex chemical reactions. As NO is consumed by this process, it is no longer available to react with ozone. When additional VOCs are added to the atmosphere, a greater proportion of the NO is oxidized to NO_2_, resulting in greater ozone formation. Anthropogenic sources of NO lead to higher levels of NO_2_ in the atmosphere, which will be available for photolysis. The formation and increase in ozone concentrations occur over a period of a few hours. Shortly after sunrise, NO and VOCs react in sunlight to form ozone. Throughout the morning, ozone concentrations increase while NO and VOCs are depleted. Eventually, either the lack of sunlight, NO, or VOCs limit the production of ozone. This diurnal cycle varies greatly depending on site location, emission sources, and weather conditions. Precursor emissions of NO and VOC are necessary for ozone to form in the troposphere. Understanding the nature of when and where ozone precursors originate may help forecasters to factor day-to-day emissions changes. For example, if a region’s emissions are dominated by mobile sources, emissions, and hence the ozone that forms may depend on the day-of-week commute patterns. The dominant NO_x_ producers are combustion processes, including industrial and electrical generation processes, and mobile sources such as automobiles. Mobile sources also account for a large portion of VOC emissions. Industries such as the chemical industry or others that use solvents also account for a large portion of VOC emissions. It is observed that the emission levels correlate well with population levels, which are larger in the eastern parts of the United States and near metropolitan areas. EPA estimations of anthropogenic emissions and annual biogenic emissions show that biogenic VOC emissions occur mostly in the forested regions of the United States (Southeast, Northeast, and West Coast regions). Biogenic VOC emissions from forested and vegetative areas may impact urban ozone formation in some parts of the country. Biogenic NO_x_ emissions levels are typically much lower than anthropogenic NO_x_ emissions levels.

Due to the importance of urban ozone as a pollutant, a number of research studies have been attempted by different research groups in the U.S. and other countries to understand the characteristics of urban ozone through observation analysis and modeling. The studies of Kleinman *et al*. [7] over New York City and Thielmann *et al*. [8] over Milan, Italy, suggest that ozone production is more sensitive to VOC than NO_x_ production. Zaveri *et al*. [9] reported in the findings from a field campaign that the downwind O_3_ concentrations in the Nashville plume are more sensitive to NO_x_ emissions than anthropogenic VOC emissions. In contrast, Kleinman *et al*. [10] suggested that O_3_ production in the high NO_x_ portions of the Philadelphia urban plume is associated with VOC. Different studies over different regions clearly indicate that the ozone formation is strongly dependent on locations due to the varied ambient chemical conditions in different regions. In addition to ambient chemical conditions, ozone concentrations in a city plume are very sensitive to meteorological conditions, including extra-urban scale transport winds, vertical structures of wind fields and mixing processes, and mesoscale convergence zones and transport processes. The influence of meteorological conditions on the transport and distribution of ozone has been studied [11,12]. A number of observations and model studies related to the Mexico City metropolitan area have reported strong influence of CO and NO_x_ emissions on the production and distribution of ozone [13–17]. A fully coupled weather-chemistry model – WRF/Chem – [18] (details are given in Section 3, is currently used by many researchers for air quality studies [19–23] and many of these are reported from Mexico City. In a study on the air quality modeling of the Houston-Galveston area with WRF/Chem, Misenis *et al*. [21] reported that the Yonsei University and Mellor-Yamada-Janjic planetary boundary layer (PBL) schemes produced better simulations of ozone for the Houston (Texas, U.S.A.) area. Tie *et al*. [24] reported that WRF/Chem simulated the time and spatial variations of ozone, but with underestimated ozone mixing ratios up to 25%. Zhang *et al*. [17] reported that the model performs much better during daytime than nighttime for both chemical species and meteorological variables over the Mexico City metropolitan area.

During the last few years, a number of research studies on air quality over the Mississippi Gulf coast region were made by the research group at TLGVRC, JSU [25–28]. Anjaneyulu *et al*. [26] observed that the main reason for high ozone concentrations in the coastal areas of Gulf Coast is the sea breeze resulting from differential heating of the land and ocean.

Anjaneyulu *et al*. [29] studied a moderately severe ozone episode with ozone values exceeding 80 ppbv over the Mississippi Gulf coast region that occurred during 8–11 June 2006. For this study, WRF/Chem model was configured with three two-way interactive nested domains (36-12-4 km resolution) and 31 vertical levels with the inner finest domain covering the Mississippi coast and several simulations were performed to study the sensitivity to planetary boundary layer and land surface processes. Their study revealed that the combination of YSU PBL and NOAH land surface schemes gave best simulations for all the meteorological and air quality fields with least bias, root mean square error and highest correlation values. Differences were evident with different combinations; as MYJ PBL producing relatively shallow mixing layers than with YSU and ACM schemes, 5-layer soil model simulating relatively deeper mixed layers than NOAH LSM, ACM PBL scheme producing localized higher concentrations.

The present study is different from the above study as the chosen area covering the Jackson metropolitan urban area is of a micro-scale nature, with the simulations being performed at a very high resolution of 1 km. The results of sensitivity experiments in the earlier study [29] were used to choose the best possible combination of the PBL and land surface schemes.

In this study, an attempt has been made to study the evolution of surface ozone and other precursor emissions like NO_x_ and CO over southeast parts of U.S. in general and the Jackson, MS urban area in particular. Jackson City is the capital and the most populous city of the U.S. state of Mississippi. It is one of two county seats of Hinds County but also contains areas in Madison and Rankin counties. According to the United States Census Bureau, the city has a total area of 106.8 square miles, of which 104.9 square miles is land, and 1.9 square miles is water and is the habitat for a population of 173,861 (628,817 in the Jackson metropolitan area). Jackson is home to several major industries, which include electrical equipment and machinery, processed food, and primary and fabricated metal products. It houses large network of roadways with Interstate Highways I-20, I-55 and I-220; U.S. Highways 49, 51 and 80; State Highways 18 and 25. We are motivated to take up this study to assess the performance of WRF/Chem in the simulation of a moderate ozone episode over an urban locality in which the atmospheric flow patterns are strongly influenced by local terrain and land cover patterns and the results from this study could provide useful information of urban air pollutants to air quality regulatory agencies and health administrators.

The details of the experiment methodology which includes WRF/Chem model, its adaptation and design of experiments are provided in Section 2; results are described in Section 3 and conclusions are given in Section 4.

## 2. Experimental Section

In this section, details of the WRF/Chem model, its adaptation to the present study, design of numerical experiments are described.

### 2.1. WRF/Chem Model

The Weather Research and Forecasting–Chemistry model (WRF/Chem) is a new generation regional air quality modeling system developed at NOAA (National Oceanic and Atmospheric Administration) [18]. The version of WRF/Chem used in this study is 3.1. Its meteorological model Advanced Research WRF (ARW) is a mesoscale weather model developed by NCAR [30] and other research institutes in the U.S. It consists of fully compressible non-hydrostatic equations, terrain following vertical coordinate and staggered horizontal grid. The model has several options for spatial discretization, diffusion, nesting, lateral boundary conditions and parameterization schemes for sub-grid scale physical processes. The physics consists of microphysics, cumulus convection, planetary boundary layer turbulence, land surface, longwave and shortwave radiation. The chemistry module of WRF/Chem treats the processes of transport, wet and dry deposition, chemical transformation, photolysis, aerosol chemistry and dynamics. The air quality component of the model is fully consistent with the meteorological component; both components use the same transport scheme, the same horizontal and vertical grids, the same physics schemes and the same time step for transport and vertical mixing. The model incorporates several different chemistry, aerosol and photolysis schemes. The model is consistent with all conservative transport done by the meteorology model. The resolution of the model is flexible, ranging from a few kilometers to hundred kilometers.

### 2.2. Model Configuration and Initialization

**Figure 1.**
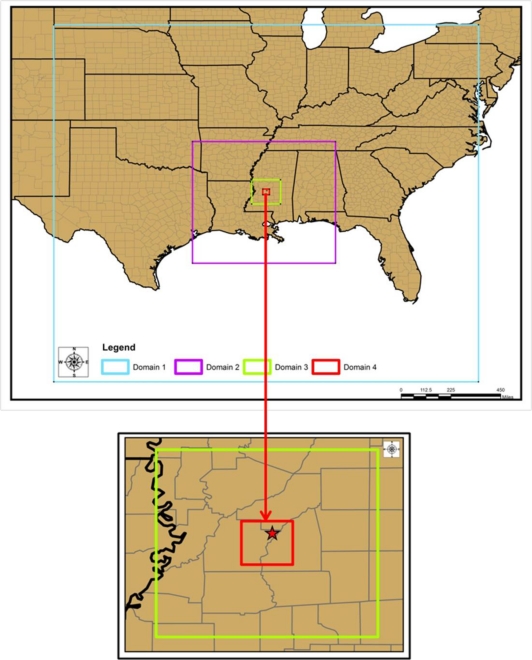
Model domains. Innermost domain covering the study region of Jackson, MS is shown as red square. Observation site is shown as star.

The WRF/Chem model is adapted to have four nested domains with horizontal resolution of 36, 12, 4 and 1 km (Figure 1). The outermost domain covers southeast parts of the U.S. and the adjoining ocean region; the second domain with 12 km resolution covers Louisiana and Mississippi states, parts of Texas, Arkansas, Florida, Alabama and Tennessee and the adjoining ocean region; the third domain with 4 km resolution covers central MS state and the innermost 1 km domain covers Hinds County.

A total number of 40 vertical levels, with 10 levels in the lower atmosphere region below 800 hPa for better resolution of the troposphere and the PBL were taken respectively. Terrain and land cover data at the resolution of ∼0.9 km as available from US Geological Survey (USGS) sources are used to interpolate to the model grid domains. The file that can be obtained at http://www.mmm.ucar.edu/wrf/src/wps_files/geog.tar.gz contains all the necessary terrain and land cover data files (30″, 2′, 5′, and 10′ resolution). The initial and lateral boundary conditions for meteorology component are taken from the National Centers for Environmental Prediction (NCEP) Final (FNL) Global Analysis data available at 1°resolution and at six hour intervals. The chemistry is initialized with idealized profiles, with the anthropogenic emissions data taken from the U.S. Environmental Protection Agency (EPA) National Emissions Inventory (NEI) inventory. This data consists of area type emissions on a structured 4-km grid and point type emissions at latitude and longitude locations. The data is interpolated to model grids using the emissions processing program available with WRF/Chem. The biogenic emissions are calculated using the scheme of Guenther *et al*. [31,32]. The model runs are conducted with the Lin microphysics [33] and new Grell convective parameterization scheme for domains 1, 2. Atmospheric shortwave and longwave radiation components are computed using the Goddard scheme [34] and RRTM scheme [35], respectively. The vertical turbulent transport in PBL is treated using the YSU non-local diffusion scheme [36]. The surface processes are computed using the Noah land surface model (NOAH) [37] which treats explicit soil and vegetation effects. The chemistry options used in the model are the Regional Acid Deposition Model version 2 (RADM2) gas-phase chemical mechanism [38,39] and Madronich photolysis scheme [40]. For the present study no aerosol module is included. The chemistry was initialized with idealized profiles. The various options used in the model are given in Table 1. The model was integrated starting from 19 CST of 5 June, 2006 (00 UTC of 6 June 2006) continuously for 48 hours. The model results for the 24 hour period from 19 CST of 6 June are only analyzed discarding the first 24 hours as warm up period.

## 3. Results and Discussion

**Figure 2.**
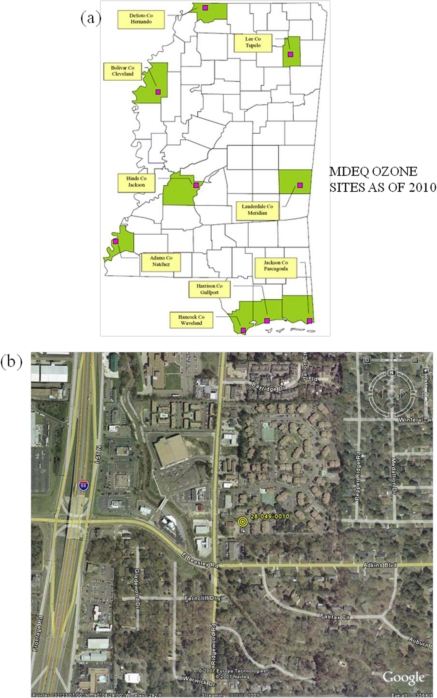
(**a**) MDEQ Observation sites of ozone in Mississippi State. (**b**) MDEQ observation site of ozone in Hinds County.

In the State of Mississippi the Mississippi Department of Environmental Quality (MEDQ) is the only agency operating an ambient air quality network and there are no other local agencies. It is noted that only one observation site located at 5810 Ridgewood Highway, Jackson, Hinds, MS with the coordinates as 32.38578 N, 90.141006 W (Figure 2a) is available for ozone measurement in Hinds County, MS, as per MDEQ (http://www.deq.state.ms.us/mdeq.nsf/page/geology_home).

**Figure 3.**
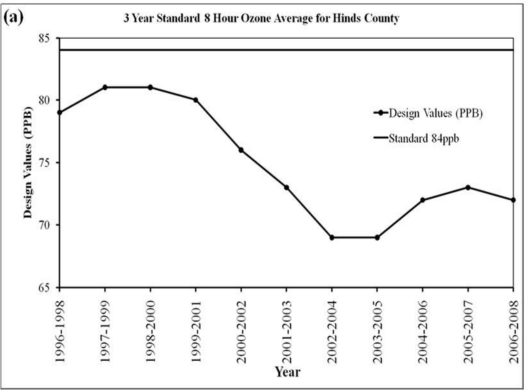

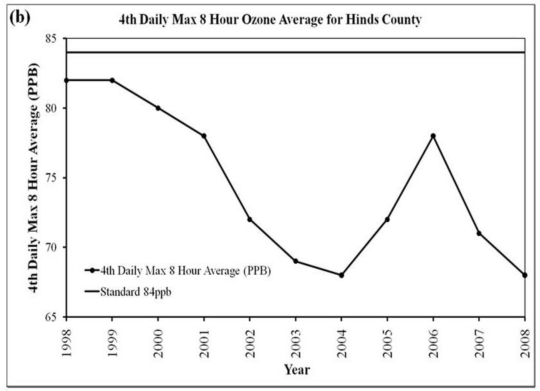
Ozone variations over Hinds County during 1996–2008 (**a**) 3 year standard 8 hour ozone average (ppb). (**b**) 4th Daily maximum 8 hour ozone average (ppb) [Mississippi Department of Environmental Quality 2008 Air Quality Data Summary].

**Figure 4.**
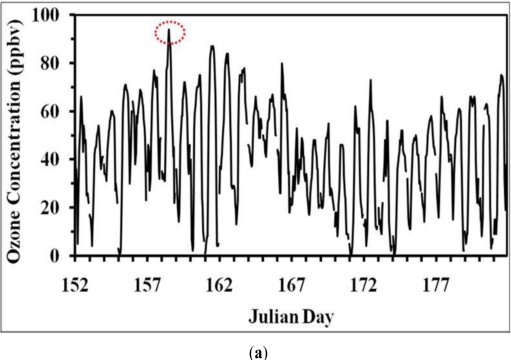

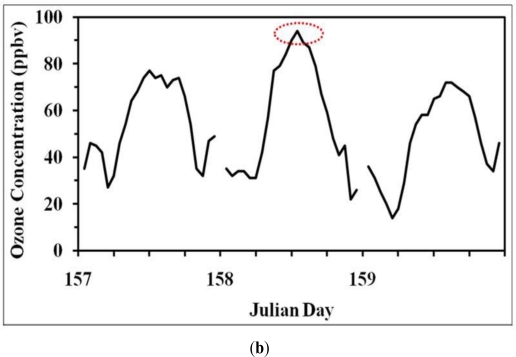
Hourly observations of ozone (ppbv) at the location in Jackson city, MS (5810, Ridgewood Highway, Jackson, Hinds, MS; 32.385758 N; 90.141006 W) **(a)** for June 2006. **(b)** 6–8 June 2006 (Julian Day 152 correspond to June 1, 2006). Red circle denotes the peak.

As per the location (Figure 2b), the observation site is situated in the urban city area of Jackson, MS and in close proximity to US-Interstate Highway (I-55), in a conglomeration of urban roadways and business premises where mobile traffic is active and continuous. The 3 year standard 8-hour ozone average for Hinds County (Figure 3) shows a maximum of 80 ppb during 1996–98 to 1999–2001, a gradual decrease to a low of 70 ppb during 2002–2005 and a slow increase thereafter. The fourth daily maximum 8-hour ozone average distribution shows a value of 82 ppb in 1998 and 1999, then decreases to reach a value of 68 in 2004, increases to 78 ppb in 2006 and decreases thereafter. These variations indicate that in 2006 there was a sudden increase of surface ozone over Hinds County. Due to this reason, the time series of ozone concentration for the year 2006 was examined and a peak concentration of 90 ppb was observed on 7th June 2006 (Figure 4 a,b). An examination of hourly time series for the 3-day period, 6–8 June 2006, indicate that the peak with maximum of 90 ppb occurred at 1 PM CDT (local time) on 7th June, as compared to maxima of 78 ppb and 75 ppb on 6 and 8 June respectively. Although this does not characterize a very severe ozone episode, it is important as the 8-hour ozone values exceeded 60 ppbv, a threshold which can seriously affect people suffering from respiratory deficiencies (as per U.S. EPA) and thus indicates a moderate severe ozone pollution for human health.

In the present study the WRF/Chem model was used to simulate the spatial and temporal variations of surface ozone and other precursor pollutants CO (Carbon Monoxide), NO (Nitric oxide), NO_2_ (nitrogen dioxide) and HONO (nitrous acid). The model was integrated continuously for 48-hours from 7 PM 5 June 2006, the outputs for the first 24-hours were discarded as associated with warm-up period, and the outputs for the 24-hour period from 7 PM CDT 6 June 2006 to 7 PM 7 June 2006 at 30 minute interval were analyzed.

**Figure 5.**
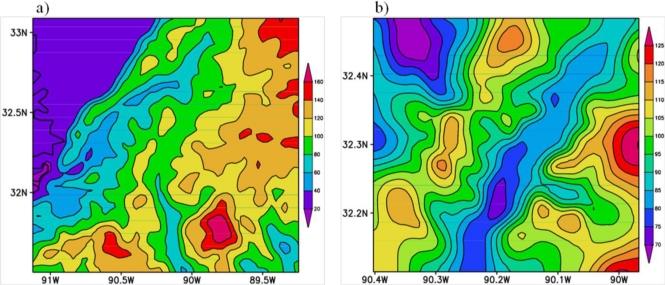
Terrain variations over (**a**) Domain 3 and (**b**) Domain 4.

**Figure 6.**
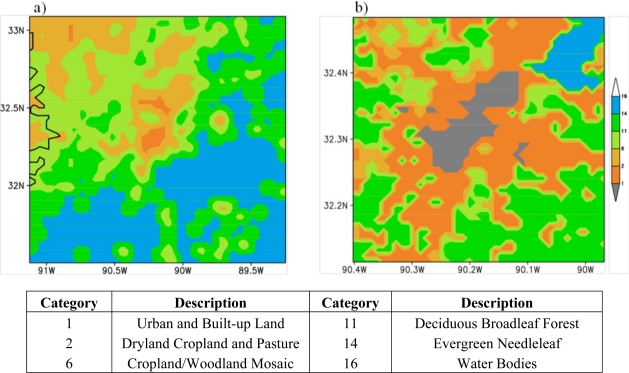
Land cover variations over (**a**) Domain 3 and (**b**) Domain 4. The description of colors for land cover is shown below the figure.

As explained in Section 3, local scale atmospheric flow patterns, especially within the PBL, are known to be influenced by the terrain features and land cover patterns as differential heating over different land cover regions and frictional effects due to terrain variations produce variations in the atmospheric flow on meso- and micro- scales. The terrain height features for domains 3 and 4 are presented in Figure 5. The central parts of MS (domain 3 region) are characterized by low elevations to the northwest and higher elevations (exceeding 100 m) on the eastern side. This means that elevated plateau is towards east and plain low-lands exist on the west side. Domain 4 region, covering Hinds County, shows that elevated terrain is situated on the east central and southeast parts low plain regions are located over central parts extending from central south to northeast regions. The land cover for the domain 3 and domain 4 are presented in Figure 6. For domain 3, the region towards most of the south and eastern parts are covered by different forest types whereas the central parts and northwest parts are demarcated as urban land. For domain 4, most of the central region is urban land with surrounding forest and ROSS BARNETT Reservoir exist towards northeast of the domain and Pearl River flows across from northeast towards south-south-west. These indicate the urban land use of the Jackson City and surroundings forms an important component of the central MS region for urban pollution studies.

**Figure 7.**
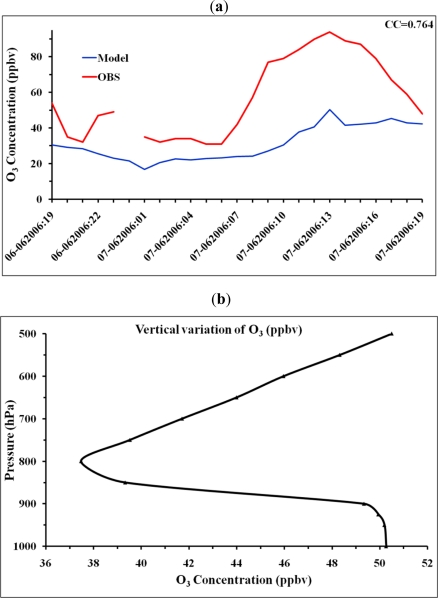
(**a**) Observed and model simulated hourly surface ozone (ppbv) at the Jackson city observation site during one day period from 7 PM 6 June to 7 PM 7 June 2006 CDT. (**b**) Model simulated vertical variation of surface ozone (ppbv) at 1 PM CDT 7 June, 2006 corresponding to the Jackson city observation site.

The time series of model simulated surface ozone for the 24 hour period from 7 PM CDT 6 June 2006 to 7 PM CDT 7 June 2006 corresponding to the observation site located at 32.38578 N, 90.141006 W are shown in Figure 7a. This time series shows the surface ozone gradually decreased from 7 PM onwards, reaching a minimum around midnight, and stayed nearly constant till dawn and gradually increased from dawn to noon, reaching a maximum around 1 PM CDT. It is noted that the model simulated a maximum of 50 ppb as compared to observational maximum of 90 ppb. The model simulated the time variations and the time of attainment of maximum same as of observations as evident from the correlation coefficient of 0.764. The underestimation of the maximum value may be due to exclusion of VOCs and use of ideal profiles. The vertical variation of the model simulated ozone at 1 PM CDT on 7 June 2006 corresponding to the observation site location (Figure 7b) shows that the magnitude of ozone is nearly constant up to 900 hPa level, decreases rapidly up to 800 hPa level and then gradually increases upward. This means that there is good mixing of O_3_ in the PBL region extended up to 900 hPa level, as is common for a summer afternoon.

**Figure 8.**
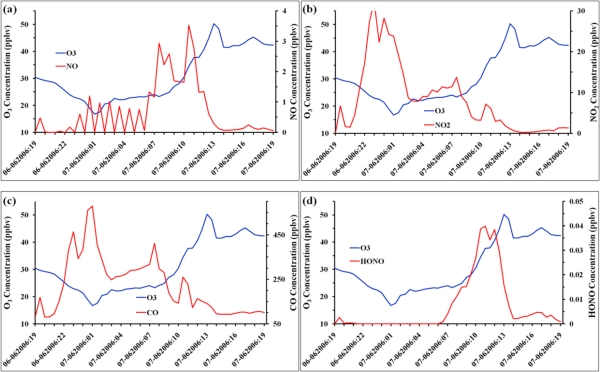
Model simulated time series (30 minute interval) of (**a**) NO (**b**) NO_2_ (**c**) CO and (**d**) HONO along with ozone corresponding to the Jackson city observation site.

The model simulated time series of the pollutant precursors – NO, NO_2_, CO, HONO – are also analyzed for the corresponding time period and presented in Figure 8. In this figure, time series of each of the pollutant is presented along with that of ozone to have an understanding of their relationship. The time series of CO shows a maximum occurring around midnight and gradually decreasing, with slight increasing trends at certain times during the daytime. The time series of NO_2_ follows the same trend as CO, with s maximum around midnight and short increasing trends during daytime. The nearly the same trends in CO and NO_2_ indicate contribution from mobile vehicles and accumulation of these pollutants in the stable night time atmosphere. The maximum during the first parts of the night could be attributed to these accumulations and decrease during later half may be due to their dispersion. The increasing trends with smaller maxima may be due to the reaction between NO with VOC leading to NO_2_ and other products. The time series of NO shows minimum values during night time and their increase from dawn attaining a maximum around 11 AM. This indicates the conversion of NO_2_ to NO in the presence of UV radiation through the mechanism given in Section 2. The occurrence of NO is maximum at 11 AM, two hours prior to ozone maximum, indicating the mechanism of NO_2_ splitting as NO and O and the formation of O_3_ through the amalgamated reaction of O and O_2_. A similar explanation holds good for CO, as O_3_ is produced in the troposphere by the photochemical oxidation of hydrocarbons in the presence of nitrogen oxides (NO and NO_2_) [41].

The time series of HONO shows a rapid increase from dawn reaching a maximum around 10 AM CDT and decreasing from noon time. The occurrence of a maximum between 10–12 AM indicates its emission from nearby combustion sources and its decrease after 12 noon could be attributed to its breakdown through reactions with NO and OH in the presence of sunlight. This indirectly supports formation of ozone as it leads production of NO and OH.

**Figure 9.**
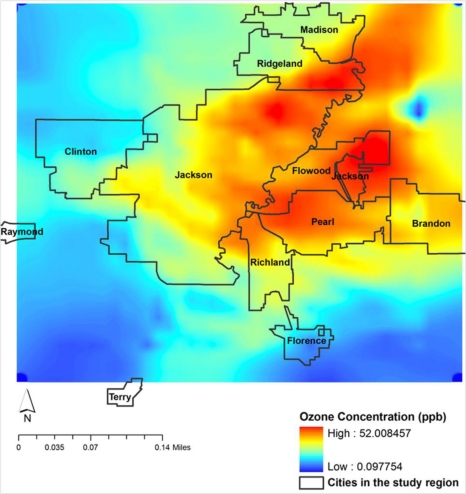
Spatial distribution of model simulated surface ozone (ppbv) over domain 4 covering Jackson city and neighbourhood at 1 PM CDT 7 June 2006.

In all, NO and HONO contribute directly to O_3_ formation in the presence of UV radiation during daytime. This is evident through the occurrence of maximum NO and HONO values 1–2 hours prior to the occurrence of the O_3_ maximum. These reactions also show the secondary maxima in CO and NO_2_ during daytime. This also shows that the chemical reactions adapted in WRF/Chem model for the present study seem to be appropriate although the magnitude of O_3_ is underestimated by 30%. Only the spatial distributions of O_3_ and other pollutants within the innermost domain covering Hinds County are presented and discussed. This is to focus the model simulations for the urban Jackson City and neighboring areas of Hinds County. The spatial distributions of O_3_ corresponding to the time of maximum (*i.e.*,) 1 PM 7 June 2006 are shown in Figure 9.

**Figure 10.**
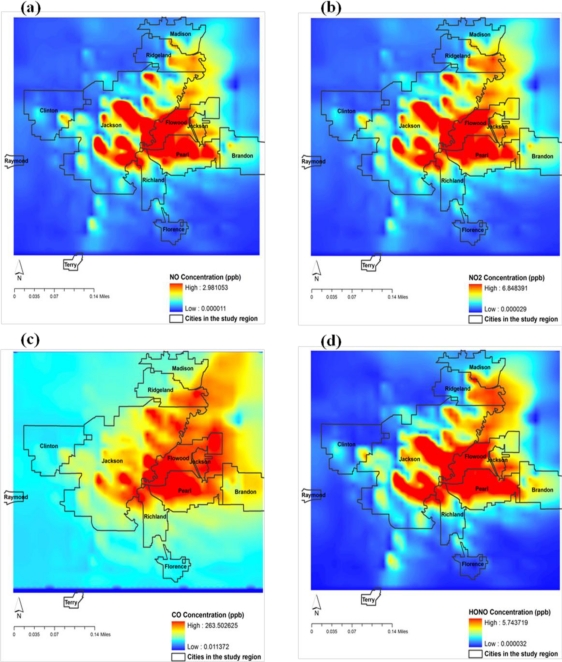
Spatial distribution of model simulated (**a**) NO (**b**) NO2 (**c**) CO and (**d**) HONO (*1e-2, ppbv) over domain 4 covering Jackson city and neighbourhood at 1 PM CDT 7 June 2006.

It is noted that the central, east and northeast parts of model domain, covering the regions of Jackson, Flowood, Pearl and parts of Ridgeland and Madison have the maximum concentrations. The western and southern parts covering Clinton, Raymond and Florence are less affected. This spatial pattern indicates the maximum as associated with the identified urban locations due to mobile traffic and combustion sources. This simulation also indicates the most vulnerable regions for pollution. The spatial distribution of NO (Figure 10a) shows higher concentrations in the central regions covering the urban Jackson city area. The maxima over the central parts of study region, identified as urban Jackson City, indicate the production of NO due to mobile traffic. However, the concentrations of NO are noted to extend towards northeast parts of domain coinciding with the maximum O_3_ regions.

A similar spatial distribution is observed for NO_2_ (Figure 10b), indicating that these maxima are due to emissions from mobile sources. The spatial distribution of CO (Figure 10c) shows maximum concentrations over eastern parts of Jackson city extending towards the northeast. This pattern is similar to those of NO and NO_2_. The spatial distribution of HONO (Figure 10d) shows concentration over central parts extending towards northeast but with higher regions towards the northwest as compared to the southeast. This pattern indicates the presence of combustion sources over central and northeast parts.

**Figure 11.**
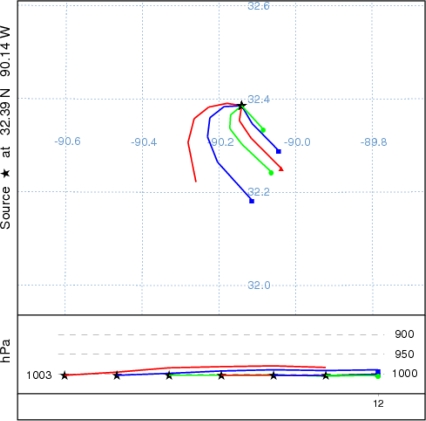
Back trajectories from the observation site drawn at 1-hour intervals starting from 1 PM CDT 7 June 2006 each with time length of 6 hours.

With a view to ascertain the possible sources of the pollutants that contribute ozone production, six back trajectories (Figure 11) were drawn at 1-hour intervals with a time length of 6-hours starting from 1 PM CDT 7 June 2006 from the observation site in Jackson, MS. This also falls in the same area where maximum ozone is depicted in Figure 9. These back trajectories indicate the air parcels originating from west and south, where the road-ways are located. From this we may presume that the pollutants mainly of mobile origin are responsible for the production of ozone in sunlight hours.

A comprehensive picture of the pattern of the spatial variations of NO, NO_2_, CO and HONO indicate the pollutant concentration over Jackson city region and that mobile and combustion sources in Jackson, Ridgeland and Madison all contributing towards the production of O_3_ through the chemical reaction mechanisms explained in the Introduction

## 4. Conclusions

- The WRF/Chem model shows the capability of simulating ozone production and its diurnal variations at a fine resolution of 1 km. This shows the potential of WRF/Chem model for urban pollution studies in contrast to the earlier usage of running separate models for urban pollution integrated with independently generated atmospheric model outputs.
- The air pollution records of the last decade show the occurrence of a moderate air pollution episode of ozone formation on 7 June 2006 with a magnitude of 90 ppbv in the study region. Prior to and after the episode the surface ozone concentrations were low on 6 and 8 June (about 70–75 ppbv).
- The model simulation has a fairly good agreement with the observed diurnal variation of ozone concentration in the PBL. The model simulated maximum levels of ozone concentration (50 ppb) at afternoon due to photochemical production of ozone associated with the NO_x_ emission. In early morning, NO_x_ has high concentration due to high mobile traffic and low PBL, while ozone concentration has a minimum concentration (about 10 ppbv), indicating a low photochemical activity condition. Thus emission of NO_x_ plays an important role on the ozone levels in the city.
- The occurrence of NO maximum around 11 AM, 2-hours prior to the occurrence of ozone maximum, indicates the splitting of NO_2_ as NO and O and the formation of ozone through reaction of O and O_2_. Similarly a maximum of CO indicates the production of ozone in the troposphere by the photochemical oxidation of hydrocarbons in the presence of nitrogen oxides. The occurrence of maximum NO and HONO levels, 1–2 hours prior to the occurrence of the O_3_ maximum, indicate their direct contribution to O_3_ formation in the presence of UV radiation.
- These results show that the chemical reactions adapted in WRF/Chem model for the present study seem to be appropriate although there is an underestimation of magnitude of O_3_. The underestimation of ozone by 30% in the present study could be due to exclusion of biogenic emissions and use of ideal profiles.
- The model simulated maximum levels of ozone and precursors NO, NO_2_, CO and HONO over Jackson city area and surrounding regions towards its northeast covering Ridgeland, Flowood, Pearl and parts of Madison and minimum over Clinton, Richland, Florence and Raymond indicating the regions that are vulnerable to stronger pollution.
- The model simulated spatial pattern of O_3_, NO, NO_2_, CO and HONO and the back trajectories indicate that the mobile sources in Jackson, Ridgeland and Madison are contributing significantly to their formation.
- The results of this study distinctly indicated the regions of higher and lesser ozone concentrations. Although the direct effects of surface ozone were not the focus of this study the health effects of surface ozone are well known. The study indirectly indicates the vulnerable urban regions within the City of Jackson area for ozone pollution.
- The present study demonstrates the applicability of WRF/Chem model to generate quantitative information at high spatial and temporal resolution for the development of decision support systems for air quality regulatory agencies and health administrators. Further studies are under progress to make sensitivity experiments with respect to photochemical processes, surface physics, radiation and vertical resolution and fine tune WRF/Chem model towards improvement of ozone simulation over urban regions.

## Figures and Tables

**Table 1.** Design of the model experiment.

**Dynamics**	**Primitive equation, non-hydrostatic**			
Vertical resolution	40 levels			
Domains	Domain 1	Domain 2	Domain 3	Domain 4
Horizontal resolution	36 km	12 km	4 km	1 km
Domains of integration	104.074° W–76.2928° W; 19.8601° N–43.2371° N	95.0053° W–85.6463° W; 27.6283° N–35.5859° N	91.1234° W–89.2472° W; 31.5017° N–33.0888° N	90.4022° W–89.9676° W; 32.1146° N–32.4836° N
Radiation	Goddard scheme for shortwave RRTM scheme for long wave			
Sea surface temperature	NCEP FNL analysis			
Cumulus convection	New Grell scheme on the outer grids domain 1and domain 2			
Explicit moisture	Lin scheme			
PBL turbulence	Hong scheme (Yonsei State University PBL)			
Surface processes	Noah LSM			
